# Supplementary material for: Mitotic chromosomes scale to nuclear-cytoplasmic ratio and cell size in Xenopus
Source: eLife. 2023 Apr 25;12:e84360. doi: 10.7554/eLife.84360 (PMC10260010; doi:10.7554/eLife.84360)
Supplement: Figure 1—source data 1. [file elife-84360-fig1-data1.zip › Figure 1-Source Data/Figure 1-Source Data_summary.docx]

**This folder contains the following source data:**

Figure 1-Source Data 1.csv (dataframe used to make plots for Figure 1)

- Includes published data on interphase nuclei from Jevtić and Levy, *Current Biology* 2015.
