# Supplementary material for: Mitotic chromosomes scale to nuclear-cytoplasmic ratio and cell size in Xenopus
Source: eLife. 2023 Apr 25;12:e84360. doi: 10.7554/eLife.84360 (PMC10260010; doi:10.7554/eLife.84360)
Supplement: Figure 2—source data 1. [file elife-84360-fig2-data1.zip › Figure 2-Source Data/Figure 2-Source Data_summary.docx]

**This folder contains the following source data:**

Figure 2- Source Data 1.csv (dataframe used to make plots for Figure 2)

Figure 2- Source Data 2.csv (dataframe used to make plots for Figure 2- figure supplement 1)
