# Supplementary material for: Mitotic chromosomes scale to nuclear-cytoplasmic ratio and cell size in Xenopus
Source: eLife. 2023 Apr 25;12:e84360. doi: 10.7554/eLife.84360 (PMC10260010; doi:10.7554/eLife.84360)
Supplement: Figure 3—source data 1. [file elife-84360-fig3-data1.zip › Figure 3-Source Data/Figure 3-Source Data_summary.docx]

**This folder contains the following source data:**

Figure 3-Source Data 1.csv (all data used to make plots in Figure 3B, 3D, Figure 3—supplement 3)

Figure 3-Source Data 2.csv (all data used to make plot in Figure 3—figure supplement 1)

Figure 3-Source Data 3.csv (all data used to make plots in Figure 3—figure supplement 2A)

Figure 3-Source Data 4.csv (all data used to make plots in Figure 3—figure supplement 2B)

Figure 3-Source Data 5.csv (all data used to make plots in Figure 3—figure supplement 4)

Figure 3-Source Data 6.csv (all data used to make plots in Figure 3—figure supplement 5, 6)
