# Supplementary material for: Mitotic chromosomes scale to nuclear-cytoplasmic ratio and cell size in Xenopus
Source: eLife. 2023 Apr 25;12:e84360. doi: 10.7554/eLife.84360 (PMC10260010; doi:10.7554/eLife.84360)
Supplement: Figure 4—source data 1. [file elife-84360-fig4-data1.zip › Figure 4-Source Data/Figure 4-Source Data_summary.docx]

**This folder contains the following source data:**

Figure 4-Source Data 1.svg (entire, uncropped Hi-C maps of chromosome 4L, a portion of which was displayed in Figure 4A)

Figure 4-Source Data 2.csv (all data used to construct the plot in Figure 4B)

Figure 4-Source Data 3.csv (all data used to construct the plot in Figure 4C)

Raw Hi-C data can be accessed via GEO with the token GSE217111 and password yxavasoglngrvmj.
