# Supplementary material for: Mitotic chromosomes scale to nuclear-cytoplasmic ratio and cell size in Xenopus
Source: eLife. 2023 Apr 25;12:e84360. doi: 10.7554/eLife.84360 (PMC10260010; doi:10.7554/eLife.84360)
Supplement: Figure 5—source data 1. [file elife-84360-fig5-data1.zip › Figure 5-Source Data/Figure 5-Source Data_summary.docx]

**This folder contains the following source data:**

Figure 5-Source Data 1 (all data used to generate plots in Figure 5B-C, Figure 5-figure supplement 1 and 2)

Figure 5-Source Data 2 (all data used to generate plot in Figure 5D)

Figure 5-Source Data 3 (all data used to generate plot in Figure 5E)

Figure 5-Source Data 4 (all data used to generate plot in Figure 5-figure supplement 3A)

Figure 5-Source Data 5 (all data used to generate plot in Figure 5-figure supplement 3B)

Figure 5-Source Data 6 (all raw uncropped images and analysis of Western blot in Figure 5-figure supplement 4A)

- Figure 5-Source Data 6A (folder containing all raw uncropped images, grouped by Antibody and intensity of scan)
- Figure 5-Source Data 6B (annotated Western blot, related to Figure 5-Source Data 6A)
- Figure 5-Source Data 6C (Quantification of signal intensity, normalized for the highest intensity band for each frog and each stage, performed in FIJI)

Figure 5-Source Data 7 (all data used to generate plot in Figure 5-figure supplement 4B)
