# Supplementary figures and images for: Mitotic chromosomes scale to nuclear-cytoplasmic ratio and cell size in Xenopus
Source: eLife. 2023 Apr 25;12:e84360. doi: 10.7554/eLife.84360 (PMC10260010; doi:10.7554/eLife.84360)

# High intensity scan

x-CAP-G

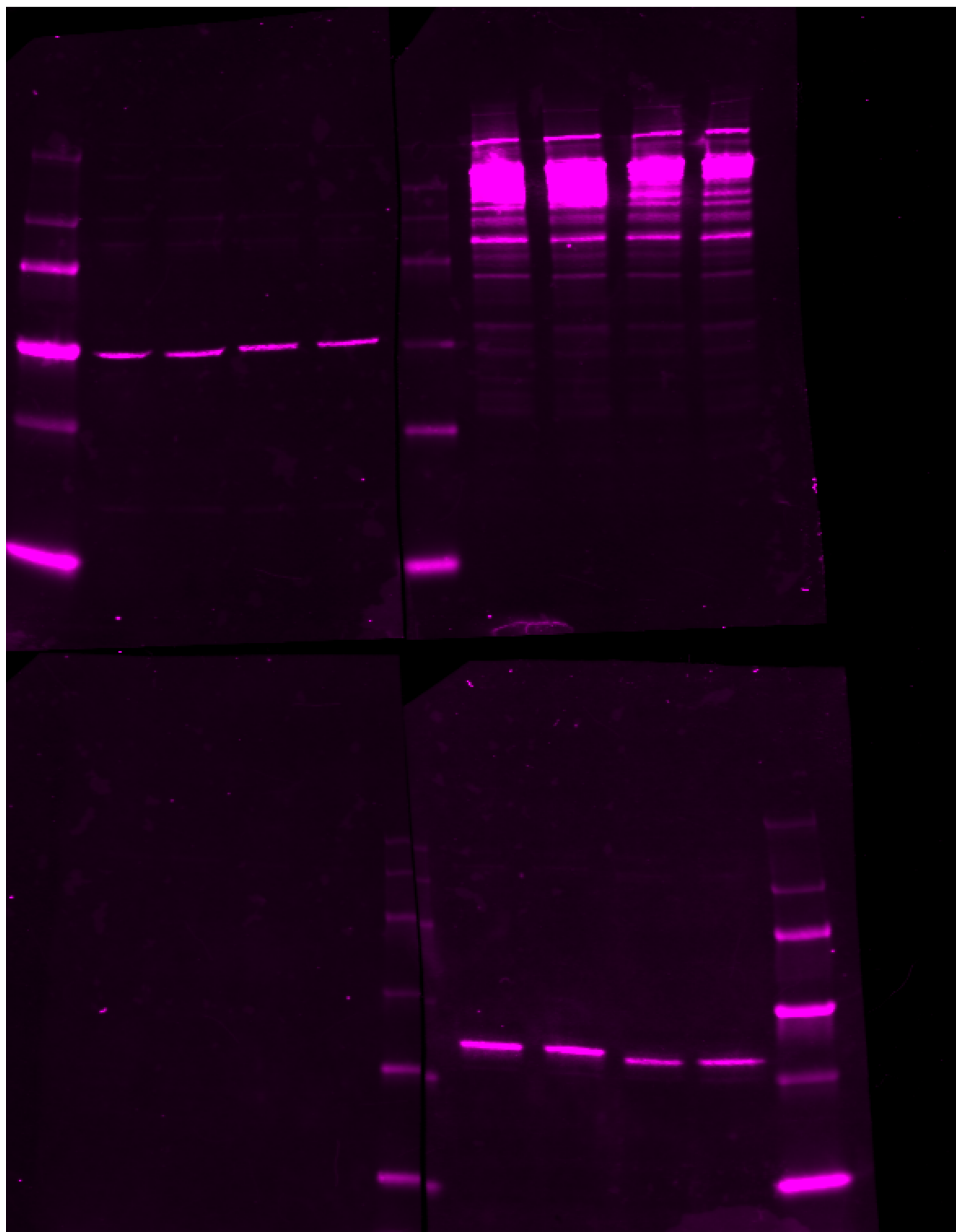

Topo2

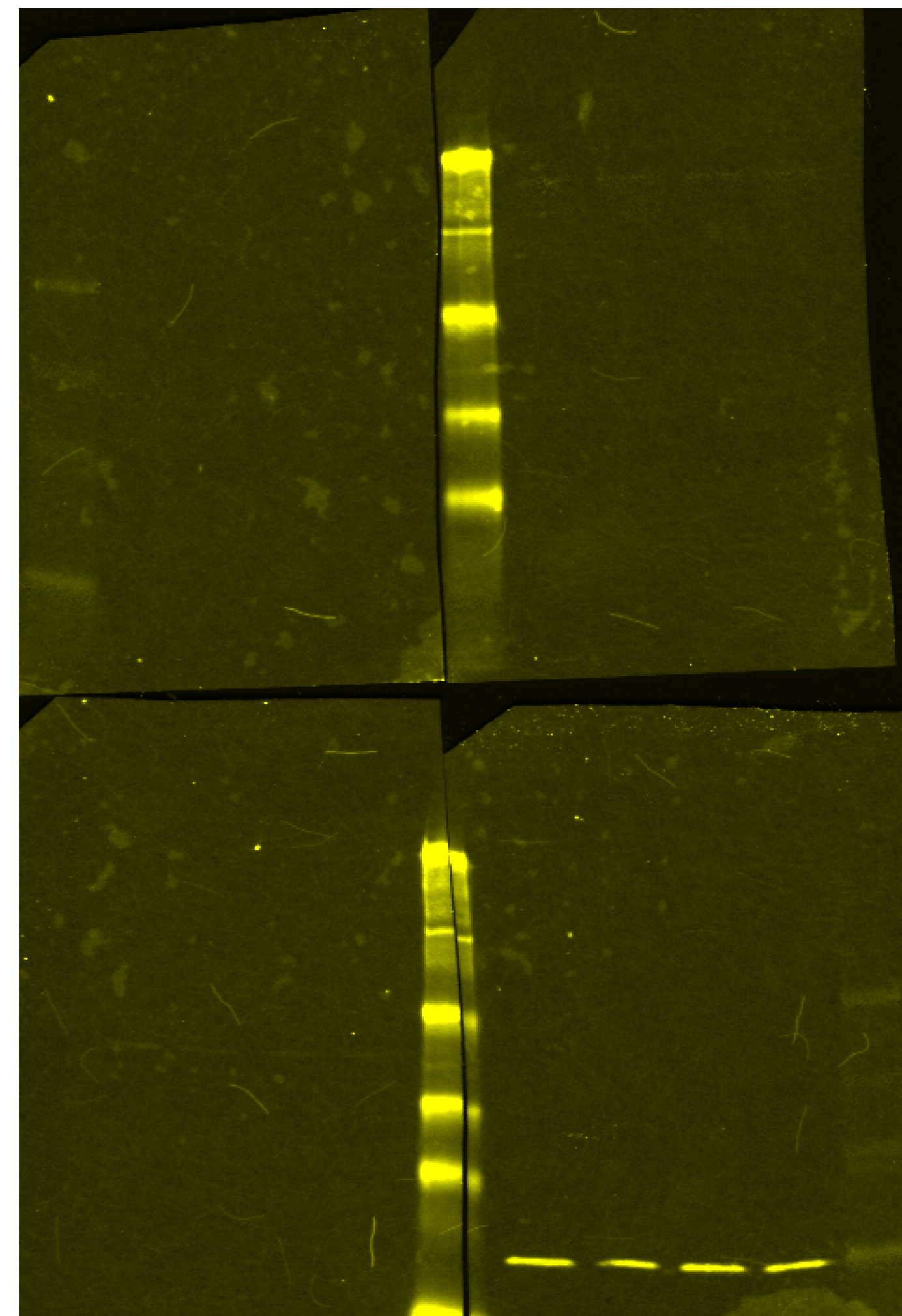

H3

# Low intensity scan

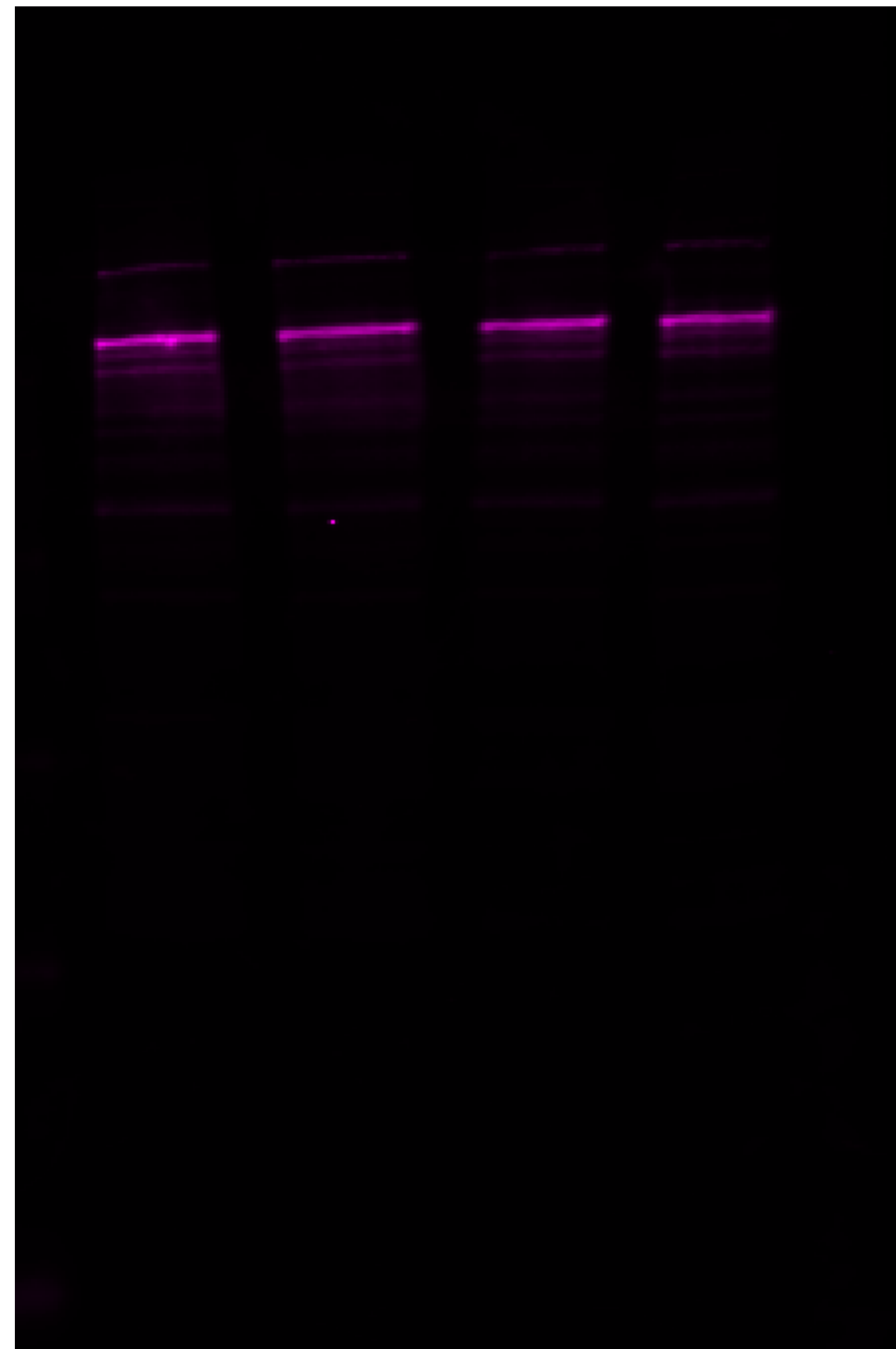

Topo2

Supplement: Figure 5—source data 1. [file elife-84360-fig5-data1.zip › Figure 5-Source Data/Figure 5-Source Data 6/Figure 5-Source Data 6B.pdf]
