# Supplementary material for: Mitotic chromosomes scale to nuclear-cytoplasmic ratio and cell size in Xenopus
Source: eLife. 2023 Apr 25;12:e84360. doi: 10.7554/eLife.84360 (PMC10260010; doi:10.7554/eLife.84360)
Supplement: Figure 6—source data 1. [file elife-84360-fig6-data1.zip › Figure 6_Source Data/Figure 6-Source Data_summary.docx]

**This folder contains the following source data:**

Figure 6-Source Data 1.csv (dataframe used to make plot in Figure 6C)

Figure 6-Source Data 2.csv (dataframe used to make plot in Figure 6D, Figure 6—figure supplement 1)

Figure 6-Source Data 3.csv (dataframe used to make plot in Figure 6F)
